# Circulating microRNA-197-3p as a potential biomarker for asbestos exposure

**DOI:** 10.1038/s41598-021-03189-9

**Published:** 2021-12-14

**Authors:** Francesca Frontini, Ilaria Bononi, Elena Torreggiani, Giulia Di Mauro, Elisa Mazzoni, Mariarita Stendardo, Piera Boschetto, Roberta Libener, Roberto Guaschino, Federica Grosso, Giovanni Guerra, Fernanda Martini, Mauro Tognon

**Affiliations:** 1 Laboratories of Cell Biology and Molecular Genetics, Section of Experimental Medicine, Department of Medical Sciences, School of Medicine, University of Ferrara, 44121 Ferrara, Italy; 2 Department of Translational Medicine and for Romagna, University of Ferrara, 44121 Ferrara, Italy; 3 Department of Medical Sciences, Unit of Occupational Medicine, University of Ferrara, 44121 Ferrara, Italy; 4 Department of Integrated Activities Research and Innovation - SS. Antonio and Biagio and Cesare Arrigo Hospital, 15121 Alessandria, Italy; 5 Blood Transfusion Centre, City Hospital, 15121 Alessandria, Italy; 6 Mesothelioma Unit - SS. Antonio and Biagio and Cesare Arrigo Hospital, 15121 Alessandria, Italy; 7 Clinical Laboratory Analysis, University Hospital of Ferrara, 44121 Ferrara, Italy; 8 Clinical Laboratory Analysis, City Hospital of Bergamo, 21127 Bergamo, Italy

**Keywords:** Cell biology, Molecular biology, Biomarkers, Medical research, Risk factors

## Abstract

Asbestos is considered the main cause of diseases in workers exposed to this mineral in the workplace as well as an environmental pollutant. The association between asbestos and the onset of different diseases has been reported, but asbestos exposure specific biomarkers are not known. MicroRNAs (miRNAs) are small, single-strand, non-coding RNAs, with potential value as diagnostic, prognostic, and predictive markers in liquid biopsies. Sera collected from workers ex-exposed to asbestos (WEA) fibers were compared with sera from healthy subjects (HS) of similar age, as liquid biopsies. The expression of the circulating miRNA 197-3p was investigated employing two different highly analytical PCR methods, i.e. RT-qPCR and ddPCR. MiR-197-3p levels were tested in sera from WEA compared to HS. MiR-197-3p tested dysregulated in sera from WEA (n = 75) compared to HS (n = 62). Indeed, miR-197-3p was found to be 2.6 times down-regulated in WEA vs. HS (p = 0.0001***). In addition, an inverse correlation was detected between miR-197-3p expression level and cumulative asbestos exposure, being this miRNA down-regulated 2.1 times in WEA, with high cumulative asbestos exposure, compared to WEA with low exposure (p = 0.0303*). Circulating miR-197-3p, found to be down regulated in sera from WEA, is proposed as a new potential biomarker of asbestos exposure.

## Introduction

Asbestos is a natural mineral^1^ of known oncogenic properties. Asbestos fibers are found in nature with different chemical compositions and physical forms^1^.

Since it is considered indestructible, this natural mineral has been used and distributed worldwide. Asbestos has been employed mainly in heat resistant artifacts. Due to its insulation characteristic, it has been used extensively in shipyards and construction. Exposure in the workplace has been significant for asbestos miners and millers, as well as for those exposed during secondary manufacturing uses. These workers were historically subject to very heavy inhalation exposure, as a result of mixing and spraying asbestos on the job^2^. Although asbestos has been banned in many countries, it should be recalled that in some areas, mainly in the developing world, its use remains unregulated^3^.

Inhaled asbestos fibers cause serious pathologies of the respiratory system, such as asbestosis, interstitial fibrosis, malignant lung and laryngeal cancers, malignant pleural mesothelioma (MPM), and neoplasms of other organs, i.e., peritoneal, pericardial, and testicular vaginal tunic mesothelioma, as well as malignant tumors of the ovary^4^. Asbestos related diseases arise mainly in workers exposed to the mineral in the workplace^5^. Asbestos fibers trapped between the pleural layers and the wall of the chest cavity induce oxidative stress and chronic inflammation, thus promoting a potential disease/carcinogenesis process^6^. It has been reported that asbestos is cytotoxic, clastogenic and mutagenic, whereas in human cells it induces specific structural and numerical chromosomal aberrations, as well as oncogene and tumor suppressor gene mutations, including K-RAS and p53, respectively^7^. Of all asbestos-related diseases, malignant pleural mesothelioma (MPM) onset may occur in workers ex-exposed to asbestos (WEA), even decades after inhalation of the fibers of this natural tumorigenic mineral^8^.

In this context, it should be recalled that asbestos has become an environmental pollutant, too, affecting subjects/patients who previously were not open to exposure in the workplace^9^. The association between asbestos exposure and the onset of different diseases has been reported by many investigators^10^.

Several publications based on toxicological investigations have pointed to the risk of respiratory disease onset being associated with fiber dimensions. Phase contrast microscopy (PCM) and transmission electron microscopy (TEM) analyses carried out in relation to fiber dimensions have indicated that this asbestos parameter is significant in assessing the risk of respiratory disease onset among WEA cohorts^11^. Such risks, including MPM, were also investigated in subjects who had been exposed to asbestos fibers in non-occupational environments, such as in the household and residential areas^9^. These investigations indicate that the risk of MPM onset from non-occupational asbestos exposure is related to the fiber-type. To date, no known factor can predict disease onset/evolution with certainty.

Liquid biopsies represent promising samples for analysis in identifying asbestos exposure markers. Indeed, until now no single biomarker has yet been reported. Indeed, the identification of specific, sensitive biomarkers, providing early, effective diagnosis in high-risk subjects is of paramount interest.

MicroRNAs (miRNAs) are small, single-strand, non-coding RNAs, with potential value as diagnostic, prognostic, predictive markers for different diseases. Many studies have indicated that miRNA expression is altered in cells and body fluids from patients affected by different diseases, while miRNAs detected in biological samples are stable and quantifiable. These data suggest that miRNAs isolated from liquid biopsies, such as serum/blood samples, can be employed as promising disease biomarkers, which include different pathologies springing from asbestos exposure^12,13^. To date, specific biomarkers of asbestos exposure have been poorly investigated.

In a previous comparative study, carried out on sera with a small-sized sample from MPM, WEA and HS cohorts, three miRNAs, i.e., miR-197-3p, miR-1281 and miR 32-3p, were found to be dysregulated using two relative techniques, such as microarray and RT-qPCR^14^. Herein, in order to undertake a significant statistical analysis, a new larger-sized sample of sera was investigated with novel absolute quantitative techniques. MiR-197-3p appears as the most promising microRNA to be analyzed. Indeed, preliminary data indicated that miR 197-3p was the only miR out of three found to be expressed in all sera analyzed.

In this investigation, sera collected from workers who had previously been exposed to asbestos (WEA) fibers were employed in our study, alongside sera from healthy subjects (HS), as liquid biopsies. In order to find a specific miRNA to be used as a biomarker for asbestos exposure sera were analyzed by quantitative PCR (qPCR) methods, such as Real-Time qPCR (RT-qPCR) and droplet digital PCR (ddPCR) techniques. It should be recalled that droplet digital PCR (ddPCR) is an improvement on conventional PCR technique, as it allows a more sensitive, accurate, absolute quantification of target nucleic acids, even when present in low target copies. MiR-197-3p was analyzed in this study using both qPCR techniques on differing circulating miRNAs from a large sample size of sera, which included WEA (n = 75) and HS (n = 62).

## Methods

### Serum samples

Serum samples from WEA (n = 75) were collected by two reference centers of the post-occupational medical surveillance program for asbestos workers, supported by the Italian Ministry of Health (i) the "Workplace prevention, hygiene and safety service", of Dolo and (ii) the Occupational Medicine Unit, University of Ferrara, Italy. WEA participated on a voluntary basis and excluded if affected by MPM. Sera from HS (n = 62) were collected at the Clinical Laboratory Analysis of the University Hospital of Ferrara, Italy, from individuals who were admitted for a routine check-up. Both cohorts’ subjects are of similar age (WEA 66.3 + 6.6 and HS 65.3 + 17.0 years + SD). All subjects signed an informed consent and the County Ethical Committee, Ferrara, Italy, approved the project (n. 151,078). All sera were obtained with the same procedure and coded anonymously with indications of age and gender. Clinicopathological characteristics, occupational and non-occupational information, were collected from the WEA cohort (Tables 1 and 2).

**Table 1:** Exposition data of the WEA cohort.

Exposition characteristics (unit of measure)	Range or diagnosis	N (%)
Cumulative asbestos exposure fibers (Asbestos fibers/cm^3^ of air, ff/cc)	Range 1: 0–8	21 (28.0) *
	Range 2: 8.1–55	21 (28.0) *
	Range 3: > 55	22 (29.3)
	N/A	11 (14.7) ^†^
Asbestos exposure (years)	Range 1: 0–20	14 (18.7)
	Range 2: 21–30	24 (32.0)
	Range 3: > 30	26 (34.7)
	N/A	11 (14.7) ^†^
Time since last previous asbestos exposure (years)	Range 1: 0–10	14 (18.7)
	Range 2: 11–20	25 (33.3)
	Range 3: 21–30	17 (22.7)
	Range 4: > 30	8 (10.7)
	N/A	11 (14.7) ^†^
Tobacco smoking status (Smoker/Ex/Non)	Smoker	11 (14.7)
	Ex-smoker	30 (40.0)
	Non-smoker	19 (25.3)
	N/A	15 (20.0) ^‡^
Tobacco smoking amount (Pack/Year, P/Y)	Range 1: 0	23 (30.7)
	Range 2: 0.1–10	16 (21.3)
	Range 3: 10.1–20	13 (17.3)
	Range 4: > 20	12 (16.0)
	N/A	11 (14.7) ^†^
Asbestosis or other asbestos related pathologies (Pathologies)	Pleural thickening	2 (2.7)
	Pleural plaques	3 (4.0)
	Fibrosis	1 (1.3)
	Bronchiectasis	1 (1.3)
	Emphysema	1 (1.3)
	Not related to asbestos	54 (72.0)
	N/A	13 (17.3) ^§^

*p < 0.05 compared to Range 3.

^†^Data available for 64 out of 75 (85.3%) subjects. ^‡^Data available for 60 out of 75 (80%) subjects.

^§^Data available for 62 out of 75 (82.7%) subjects.

**Table 2:** Clinicopathological characteristics of the WEA cohort.

Diseases, not related to asbestos	Yes/no	N (%)
Musculoskeletal diseases (yes/no)	Yes	7 (9.3)
	No	57 (76.0)
	N/A	11 (14.7)*
Cardiovascular diseases (yes/no)	Yes	29 (38.7)
	No	35 (46.7)
	N/A	11 (14.7) *
Gastrointestinal diseases (yes/no)	Yes	10 (13.3)
	No	54 (72.0)
	N/A	11 (14.7) *
Endocrine diseases (yes/no)	Yes	20 (26.7)
	No	44 (58.7)
	N/A	11 (14.7) *
Respiratory diseases (yes/no)	Yes	15 (20.0)
	No	49 (65.3)
	N/A	11 (14.7) *
Neurological diseases (yes/no)	Yes	5 (6.7)
	No	59 (78.7)
	N/A	11 (14.7)*
Genitourinary diseases (yes/no)	Yes	11 (14.7)
	No	53 (70.7)
	N/A	11 (14.7)*
Neoplastic diseases (yes/no)	Yes	10 (13.3)
	No	54 (72.0)
	N/A	11 (14.7)*
Therapy (yes/no)	Yes	57 (76.0)
	No	18 (24.0)
	N/A	0

*Data available for 64 out of 75 (85.3%) subjects.

### RNA extraction and retro-transcription (RT)

Total RNA, including miRNAs, was extracted from 200 μl of serum using the miRNeasy Mini Kit (Qiagen Cod. 217004) according to the manufacturer's protocol. RNA was eluted in 30 µl RNAse-free water and retro-transcribed using the miRCURY LNA RT kit (Qiagen Cod. 339340) following the manufacturer’s protocol. RT products were stored at − 20 °C until time of qPCR analyses. Before amplification, cDNA was diluted 1:60 to avoid interference between the two enzyme mixes^14^.

### MiRNA quantification, calibration and control

Hsa-miR-197-3p concentrations were analyzed by qPCR techniques using the specific miRCURY LNA miRNA PCR Assay (Qiagen Cod. 339306). Standard curves were generated thought 1:10 serial dilutions for hsa-miR-197-3p, using Specific synthetic Locked Nucleic Acid (LNA) oligonucleotides (Qiagen Cod. 339306, ID: YP00204380). Stock solutions (100 μM) of synthetic oligonucleotides were prepared according to the manufacturer’s protocol. These standards were used for absolute quantification of miRNAs by RT-qPCR^15^, and as positive controls in ddPCR.

### Analysis of miRNA by PCR techniques

RT-qPCR reactions were performed using the miRCURY LNA SYBR Green PCR Kit (Qiagen Cod. 339346) according to the manufacturer's instructions. Standard curves were run in parallel with samples to perform an absolute quantification of miR-197-3p.

LNA-based miRNA-specific primers were used in ddPCR with the QX200ddPCR System (Bio-Rad). A no template control (NTC), a negative control for each reverse transcription reaction (RT-neg) and a positive control were included for each PCR assay.

QuantaSoft software was used to determine specific intensity thresholds for the assay and results were exported to a spreadsheet for further analysis.

### Statistical analysis

Statistical analyses were performed using Prism 8.0 statistical software (GraphPad software, La Jolla, CA, USA). Data were analyzed using ANOVA, t-test, with or without Welch’s correction, and Chi-square^16^. If distribution was abnormal, the Kruskal–Wallis test and Mann–Whitney test, were employed. Receiver Operating Characteristic (ROC) analysis was used to assess the accuracy of RT-qPCR and ddPCR results. Moreover, linear regression model and multiple regression analysis were performed. P value < 0.05 was considered as statistically significant.

### Institutional review board statement

The study was conducted according to the guidelines of the Declaration of Helsinki. The Ethics Committee of Ferrara approved the study (Authorization number 160986).

Al subjects gave their written informed consent.

## Results

### MicroRNA analysis

**Figure 1:**
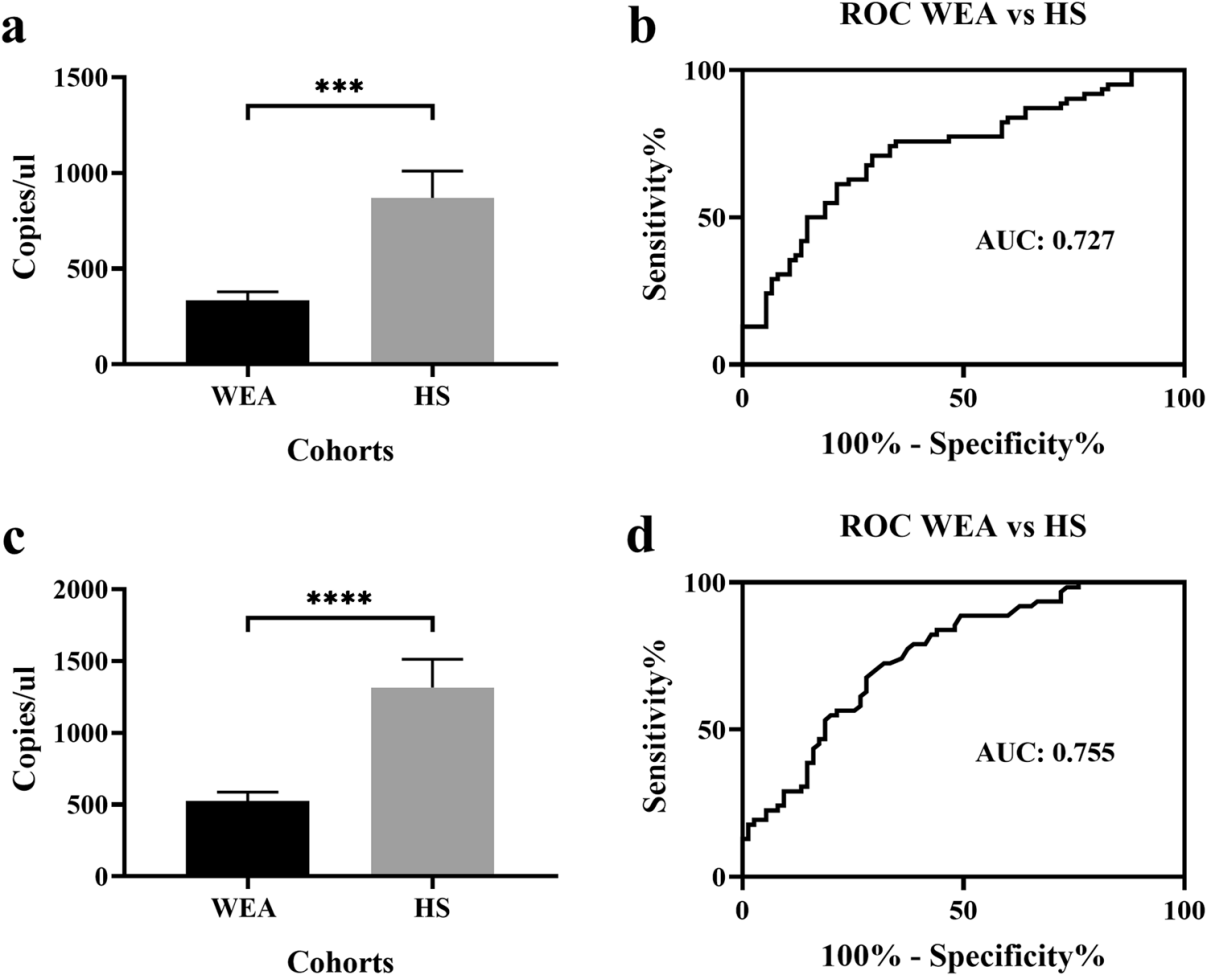
The miR-197-3p quantification. (**a**) miR-197-3p quantification in sera from WEA and HS cohorts using RT-qPCR. (**b**) ROC curve to quantify RT-qPCR accuracy. (**c**) miR-197-3p quantification in sera from WEA and HS cohorts using dd-PCR. (**d**) ROC curve to quantify dd-PCR accuracy.

In a previous study, carried out with a small sample size of sera^14^ three miRNAs, i.e., miR-197-3p, miR-1281 and miR 32-3p, were found to be dysregulated. In the present study, in order to reach statistically significant data, a large-sized sample of sera was investigated employing novel absolute quantitative methods, such as RT-qPCR and ddPCR techniques. Herein, miR-197-3p was investigated since it appears to be a promising circulating biomarker. Indeed, preliminary data indicated that miR-197-3p, out of the three dysregulated miRs, was found as expressed in all analyzed samples, which included WEA (n = 75) and HS (n = 62) (see below). In the first step of our study, RT-qPCR detected miR-197-3p in all analyzed sera (n = 137). A significantly different quantification of miR-197-3p was revealed in the two cohorts, i.e., WEA and HS (ANOVA: p = 0.0009***). Specifically, a mean of 336.0 copies of miR-197-3p/ul was detected in the WEA cohort. This quantity is significantly lower than in the HS group (870.1 copies/ul cDNA, t-test: p = 0.0001***). Hence, miR-197-3p was found to be down-regulated 2.6 times in WEA vs. HS (Fig. 1a). Receiver Operating Characteristic (ROC) analysis was used to quantify the accuracy of RT-qPCR results in discriminating data in significant comparisons. The ROC AUC for the comparative analysis of miR-197-3p levels in WEA vs. HS was 0.727 (p < 0.0001***; 95% confidence interval 0.641 to 0.813) (Fig. 1b). These ROC analyses indicate that miR-197-3p levels significantly discriminate WEA from HS. Subsequently, miR-197-3p was analyzed in the same sera (n = 137) using ddPCR. MiR-197-3p was detectable in all analyzed samples (n = 137). DdPCR analysis showed significant miR-197-3p dysregulation in the WEA cohort compared to HS (ANOVA: p = 0.0004***). A mean of 525.0 copies of miR-197-3p/µl was detected in WEA. Its serum level was significantly lower than HS (1316 copies/µl cDNA, t-test: p < 0.0001****). Hence, miR-197-3p was down-regulated 2.5 times in WEA vs. HS (Fig. 1c). ROC analysis was used to quantify the accuracy of ddPCR results in discriminating data in significant comparisons. The ROC AUC for miR-197-3p in the comparison between WEA vs. HS was 0.755 (p < 0.0001***; 95% confidence interval 0.675 to 0.835) (Fig. 1d).

**Figure 2:**
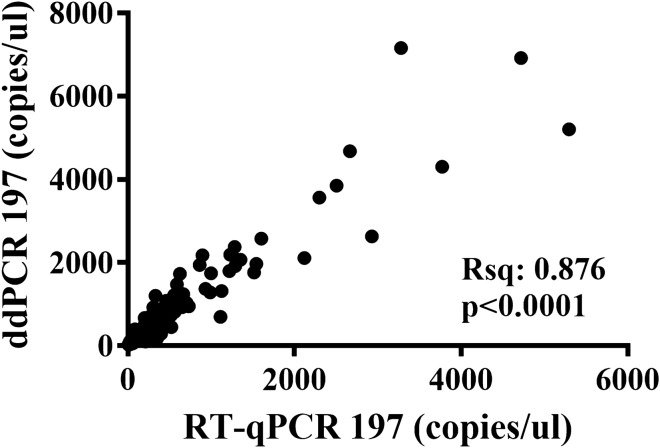
Linear regression. Linear regression analysis for the correlation between qPCR and ddPCR values.

Linear regression analysis indicated a significant correlation between qPCR and ddPCR values. The R-square was 0.900 for miR-197-3p (Fig. 2).

### Correlation studies between miRNA expression and clinicopathological characteristics for WEA

**Figure 3:**
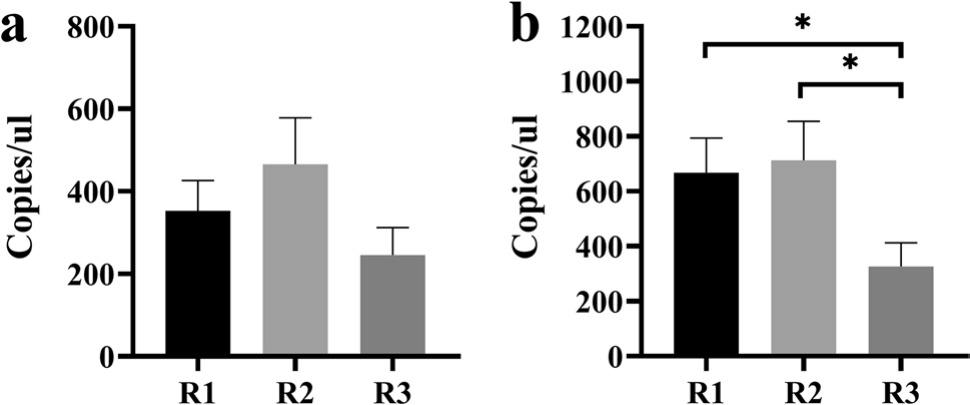
Correlation between miR-197-3p and cumulative asbestos exposure. Correlation between miR-197-3p sera expression and cumulative asbestos exposure in WEA cohort highlighted by RT-qPCR (**a**) and by dd-PCR (**b**).

Clinicopathologic characteristics, anamnestic, occupational and non-occupational information were collected for the WEA cohort (Tables 1 and 2) and studied to look for any possible correlation with miR-197-3p expression. Spearman correlation coefficients were calculated between the miRNA quantity and the evaluated independent variables. An inverse correlation was observed between miR-197-3p and cumulative asbestos exposure in the WEA cohort (Table 1). This cohort was divided into three subgroups according to the level of cumulative asbestos exposure, as follows: range 1 (0–8 asbestos fibers/cm3 of air, ff/cc), range 2: 8–55 ff/cc, range 3: for higher than 55 ff/cc exposure. Absolute miR-197-3p quantification by RT-qPCR in WEA sera detected a mean of 352.7 copies/ul in range 1; 465.9 copies/ul in range 2 and 245.9 copies/ul, in range 3, but the differences were not statistically significant (p > 0.05) (Fig. 3a). These data were confirmed and extended using ddPCR analysis, which showed two significant differences. Indeed, ddPCR revealed the following quantity of miR-197-3p in WEA sera: a mean of 667.8 copies/µl in range 1; 712.3 copies/µl in range 2 and 326.2 copies/µl in range 3. The quantity of miR-197-3p in WEA sera in range 3, revealed by ddPCR, is statistically different from range 1 (p = 0.0297*; showing up-regulation by 2.0 times) and from range 2 (p = 0.0239*; showing up-regulation by 2.1 times) (Fig. 3b).

No other statistically significant correlation was found between miR-197-3p levels and the other clinicopathologic characteristics taken into consideration, such as (i) asbestos exposure duration, (ii) years since last previous asbestos exposure, (iii) tobacco exposure, (iv) asbestosis or other asbestos related pathologies, (v) other diseases, unrelated to asbestos and (vi) pharmacological therapies (Table 3).

**Table 3:** Correlation coefficients between miRNA expression and independent variables in the WEA cohort according to Spearman.

	miR-197-3p	
	ddPCR	RT-qPCR
Age	− 0.223	− 0.112
Gender	0.215	0.208
Smoking status	0.154	0.127
P/Y	− 0.105	− 0.077
**Asbestos exposure**		
Cumulative exposure	− 0.389*	− 0.197
Years of exposure	− 0.003	0.002
Years since last previous exposure	− 0.140	− 0.085
Asbestos related pathologies	− 0.028	0.047
**Other diseases unrelated to asbestos**		
Musculoskeletal	0.114	0.131
Cardiovascular	− 0.056	− 0.101
Gastrointestinal	− 0.091	− 0.065
Endocrine and diabetes	− 0.256	− 0.120
Respiratory	− 0.091	− 0.049
Neurological	0.129	0.099
Genitourinary	− 0.126	− 0.124
Neoplastic	0.022	0.121
Therapy	0.110	0.051

Correlation coefficients were determined according to the Spearman test. miRNA levels were expressed as copies/µl of analyzed cDNA. Correlations with p < 0.05(*) were considered statistically significant.

## Discussion

Many studies have indicated that miRNAs, which are detectable in sera/cells from subjects who have previously been exposed to toxic agents, or are affected by inflammatory and/or other disorders, could be considered promising biomarkers^13,17,18^. Identifying such sensitive and specific markers for asbestos exposure in WEA appears to be of paramount importance for this class of workers, who are at risk of developing MPM. In this study, circulating miR-197-3p was investigated as a potential biomarker for asbestos exposure. MiR-197-3p, whose gene maps in chromosome 1p13.3, has been reported as being involved in cellular processes, such as cell proliferation, apoptosis, differentiation, metastasis and drug resistance^19^. Significant miR-197-3p dysregulation has been detected in sera/cells in human diseases including tumors^20–23^. In addition, miR-197-3p has been reported as playing a pivotal role as an oncogene or tumor suppressor gene by targeting key pathways^24^. Some investigations have revealed that a miR-197-mediated CKS1B/STAT3 axis exerts a role in tumor progression, which is regulated by different genes, such as Bcl-2, c-Myc, and cyclin D1^25^.

In this study circulating miR-197-3p was investigated using two PCR techniques. Digital droplet PCR (ddPCR) is an improvement on the conventional PCR technique, as it allows for a more sensitive and accurate quantification of target nucleic acids^26–33^. DdPCR is considered as being able to overcome problems relating to potential discrepancies in PCR analyses. In particular, when compared to the traditional qPCR, the ddPCR technique exhibits some advantages: (i) ddPCR performs absolute quantification based on the principles of sample partitioning and Poisson statistics, thus overcoming normalization and calibrator issues^15,26,33^; (ii) it is relatively insensitive to potential PCR inhibitors^34–36^; (iii) it has shown increased precision and sensitivity in detecting low target copies^29,37,38^; (iv) it provides analysis results directly expressed as the number of copies of target per microliter of reaction^15,32,39^. Specifically, in this study, the classic method, i.e., RT-qPCR, was combined with the more innovative ddPCR, in order to improve results. Specifically, in this study, the classic method, i.e., RT-qPCR, was combined with the more innovative ddPCR, in order to improve the results. Our data indicates that miR-197-3p is significantly down-regulated in WEA vs. HS employing both techniques. RT-qPCR and ddPCR highlight that miR-197-3p levels are significantly lower in WEA compared to HS sera by 2.6 times and 2.5 times, respectively. Statistical analyses confirm that comparative data, i.e., WEA vs. HS, are significant. Moreover, the two ROC analyses show that this miR-197-3p is a good parameter for distinguishing the WEA cohort from the HS group. A significant correlation between qPCR and ddPCR values, as indicated by linear regression analysis, confers higher robustness to our results.

It is well known that asbestos fibers can induce alterations in the immune system, while being involved in the inflammatory process^40–42^. Different studies have reported miR-197-3p as being significantly down-regulated in inflammatory conditions^43^. In addition, cross-talk between miR-197-3p and different interleukin signaling has also been identified^23,43^. Specifically, IL22R1 and IL-17A subunits, which are involved in inflammatory responses, are among the validated target genes for miR-197-3p^43,44^. Furthermore, the demonstration that miR-197-3p directly binds to one of the key molecules in the inflammatory pathways, which is the interleukin-1beta (IL-1β) receptor, type I (IL1R1) gene, explain why miR-197-3p shows an anti-inflammatory effect in different cell types^45^. Taken together, these data indicate miR-197-3p is a significant marker/parameter to be considered in the chronic inflammation processes taking place in asbestos-exposed workers.

In addition, MiR-197-3p levels showed an inverse correlation with the cumulative asbestos exposure in the WEA cohort. This result suggests that miR-197-3p could be a potential marker of the cumulative level of exposure for the oncogenic mineral in WEA. WEA exposed to a lower number of asbestos fibers exhibit higher miR-197-3p serum levels, whereas WEA exposed to higher concentrations of asbestos showed lower amounts of miR-197-3p in their serum. This result was confirmed when both PCR techniques were employed. Interestingly, data obtained using the more analytical ddPCR indicate that differing quantities of miR-197-3p in WEA sera with low exposure compared to WEA with high asbestos exposure are statistically significant. Other clinicopathologic characteristics analyzed herein did not show statistically significant correlation with miR-197-3p levels.

Overall, the investigated circulating miR-197-3p was found to be down-regulated in WEA vs. HS and its concentration decreases with an increase in the amount of inhaled asbestos fibers. From this result it may be inferred that inhaled asbestos fibers affect the down-expression of circulating miR-197-3p. Further investigations are needed to elucidate the role of miR-197-3p in WEA and the effect of its down-regulation induced by asbestos. More information on this field could be obtained by the identification/validation of miR-197-3p target genes.

Our study indicates that miR-197-3p is a new biomarker for asbestos exposure and asbestos cumulative levels in WEA. It is worth noting that there are still a worrying lack of reliable markers for asbestos-fiber exposure. Consequently, miR-197-3p may represent a new parameter for analysis in WEA sera. Liquid biopsies can be obtained from individuals with low invasive methods. Moreover, this miRNA appears to be a quantifiable, specific and sensitive biomarker. Complimenting current follow-up analyses with a minimally invasive screening test for circulating miRNA-197-3p as a biomarker could add further relevant information on WEA subjects who are at a high-risk of developing malignant pleural mesothelioma. Thus, specific and sensitive biomarkers, such as miR 197-3p, can be employed, alongside spirometry and X-ray chest analysis, during follow-up for WEA subjects.

## Data Availability

The datasets supporting the results are available from the corresponding author on reasonable request.
